# Optic nerve thinning and neurosensory retinal degeneration in the rTg4510 mouse model of frontotemporal dementia

**DOI:** 10.1186/s40478-018-0654-6

**Published:** 2019-01-07

**Authors:** Ian F. Harrison, Rozalind Whitaker, Pietro Maria Bertelli, James M. O’Callaghan, Lajos Csincsik, Martina Bocchetta, Da Ma, Alice Fisher, Zeshan Ahmed, Tracey K. Murray, Michael J. O’Neill, Jonathan D. Rohrer, Mark F. Lythgoe, Imre Lengyel

**Affiliations:** 1 UCL Centre for Advanced Biomedical Imaging, Division of Medicine, University College London, Paul O’Gorman Building, 72 Huntley Street, London, WC1E 6DD UK; 2 UCL Institute of Ophthalmology, University College London, 11-43 Bath Street, London, EC1V 9EL UK; 3 Dementia Research Centre, UCL Institute of Neurology, University College London, National Hospital for Neurology and Neurosurgery, London, WC1N 3BG UK; 4 Centre for Experimental Medicine, The Queen’s University Belfast, Belfast, BT9 7BL UK; 5 School of Engineering Science, Simon Fraser University, 8888 University Drive, Burnaby, BC V5A 1S6 Canada; 6 Eli Lilly and Company, Erl Wood Manor, Windlesham, Surrey, GU20 6PH UK

**Keywords:** Tau, Frontotemporal dementia, Neurosensory retina, MRI

## Abstract

Visual impairments, such as difficulties in reading and finding objects, perceiving depth and structure from motion, and impaired stereopsis, have been reported in tauopathy disorders, such as frontotemporal dementia (FTD). These impairments however have been previously attributed to cortical pathologies rather than changes in the neurosensory retina or the optic nerve. Here, we examined tau pathology in the neurosensory retina of the rTg(tauP301L)4510 mouse model of FTD. Optic nerve pathology in mice was also assessed using MRI, and corresponding measurements taken in a cohort of five FTD sufferers and five healthy controls. rTg(tauP301L)4510 mice were imaged (T2-weighted MRI) prior to being terminally anesthetized and eyes and brains removed for immunohistochemical and histological analysis. Central and peripheral retinal labelling of tau and phosphorylated tau (pTau) was quantified and retinal layer thicknesses and cell numbers assessed. MR volumetric changes of specific brain regions and the optic nerve were compared to tau accumulation and cell loss in the visual pathway. In addition, the optic nerves of a cohort of healthy controls and behavioural variant FTD patients, were segmented from T1- and T2-weighted images for volumetric study. Accumulation of tau and pTau were observed in both the central and peripheral retinal ganglion cell (RGC), inner plexiform and inner nuclear layers of the neurosensory retina of rTg(tauP301L)4510 mice. This pathology was associated with reduced nuclear density (− 24.9 ± 3.4%) of the central RGC layer, and a reduced volume (− 19.3 ± 4.6%) and elevated T2 signal (+ 27.1 ± 1.8%) in the optic nerve of the transgenic mice. Significant atrophy of the cortex (containing the visual cortex) was observed but not in other area associated with visual processing, e.g. the lateral geniculate nucleus or superior colliculus. Atrophic changes in optic nerve volume were similarly observed in FTD patients (− 36.6 ± 2.6%). The association between tau-induced changes in the neurosensory retina and reduced optic nerve volume in mice, combined with the observation of optic nerve atrophy in clinical FTD suggests that ophthalmic tau pathology may also exist in the eyes of FTD patients. If tau pathology and neurodegeneration in the retina were to reflect the degree of cortical tau burden, then cost-effective and non-invasive imaging of the neurosensory retina could provide valuable biomarkers in tauopathy. Further work should aim to validate whether these observations are fully translatable to a clinical scenario, which would recommend follow-up retinal and optic nerve examination in FTD.

**Electronic supplementary material:**

The online version of this article (10.1186/s40478-018-0654-6) contains supplementary material, which is available to authorized users.

## Introduction

Frontotemporal dementia (FTD) is a neurodegenerative disorder characterised by degeneration within the frontal and temporal lobes of the brain [20, 41, 46]. The disorder presents clinically with changes in behaviour, deficits in executive function and/or language impairment, but in some cases can also manifest with a motor disorder such as corticobasal syndrome, motor neuron disease or progressive supranuclear palsy (PSP) [44]. Around 30–50% of FTD cases are familial in nature, with an autosomal dominant profile of inheritance [44], and in a subset of these cases, causative mutations in the microtubule associated protein tau (*MAPT*) gene have been identified [16]. Such mutations lead to disruption of the normal binding of tau to neuronal tubulin, resulting in pathological deposition of hyperphosphorylated tau in the form of filamentous neuronal inclusions, or neurofibrillary tangles (NFTs) [16, 39]. NFTs are also observed in other pathologies such as Alzheimer’s disease (AD), Pick’s disease, corticobasal degeneration and PSP, a group of neurological disorders collectively known as tauopathies.

Numerous reports exist of early stage tauopathy patients experiencing cognitive visual changes, including changes in colour recognition [9], impairment of spatial contrast sensitivity [15], depth perception [28], perceiving structure from motion [10, 28], and also difficulties with reading and finding objects [21]. Previously however, these defects have been attributable to the well described cortical tau pathology in the brains of patients with tauopathies rather than the result of tau pathology in the optic nerve or neurosensory retina. Responsible for converting incoming light into encoded neural activity, the neurosensory retina is a laminal structure of nuclear and neuropil plexiform layers, formed by a tripartite neuronal relay: Photoreceptors - > Bipolar cells - > Retinal Ganglion Cells (RGCs), which is modulated by two classes of interneurons, Horizontal and Amacrine cells, and supported by Müller glia. RGC axons converge in the Retinal Nerve Fibre Layer (RNFL) to form the optic nerve, which then projects to the visual centres of the brain for higher order processing of input signals. With advancing age, tau becomes expressed diffusely within the inner nuclear layer (INL), and the retinal ganglion cell layer (RGCL) of the human neurosensory retina [24]. Furthermore in ocular diseases such as glaucoma, abnormally phosphorylated tau is increased in the INL and inner plexiform layer (IPL) of the retina [17, 31, 48], an observation which has similarly been made in a mouse model of ocular hypertension induced glaucoma [8]. Based on this evidence, that tau pathology also affects the eye, we hypothesized that retinal and optic nerve changes might also be associated with the pathologies in tauopathic dementias such as FTD.

Assessments of retinal changes in mice expressing the P301S mutated human *MAPT* gene driven by the mouse Thy1.2 promoter (mThy1.2) showed increased phosphorylation of tau in the RGCL and filamentous inclusions in the retina [14] without an effect on RGC number. In transgenic mice expressing the P301L mutation in the human *MAPT* gene however, driven by the mouse prion promoter (*PRNP*), total tau and phosphorylated tau levels were shown to be elevated in the RGCL, the IPL and also the INL, accompanied by a reduction in the thickness of the INL, an effect which was more pronounced in the peripheral compared to the central retina [18]. These changes were accompanied by an increase in the number but reduction in the average size of RGCs in the transgenic mice [18]. Together these data highlight the susceptibility of the neurosensory retina to mutations associated with familial FTD, and hence highlights the possibility of retinal changes in the clinical scenario. However both of these transgenic models (*MAPT* P301S driven by mThy1.2 [14], and *MAPT* P301L driven by *PRNP* [18]) show ubiquitous expression of pathological phosphorylated tau throughout the brain, quite unlike the pathological spatial pattern of tau observed in FTD [20, 41, 46]. Therefore, in order to better understand the effects of mutations associated with familial FTD on the neurosensory retina and visual system, we studied an animal model that shows spatial patterns of tau deposition similar to that seen in clinical FTD.

In the current study we assessed retinal tau pathology in the rTg(tauP301L)4510 mouse model, which harbours the same P301L mutant form of the human *MAPT* gene previously described in the study by Ho et al. [19], but driven by the forebrain-specific calmodulin kinase II a (CaMKIIa) promoter system, which results in NFT pathology in forebrain structures and the hippocampus, reminiscent of the pattern for tau accumulation observed in FTD [20, 41, 46]. Using this animal model, we show increased phosphorylated tau expression in the RGCL, as well as a decrease in RGC numbers in the neurosensory retina associated with volumetric changes (assessed through MRI) in the optic nerve and areas of the mouse brain involved in visual processing. In addition, a cohort of FTD patients carrying the *MAPT* mutation were imaged with MRI, and we show that similar to mice, volumetric changes in the optic nerve do take place in familial FTD.

## Materials and methods

### Animals

Generation of homozygous rTg(tauP301L)4510 transgenic mice, henceforth referred to as rTg4510 mice, has been described previously [43]. The rTg4510 and litter matched wildtype mice were bred on a mixed FVB/NCrl +129S6/SvEvTa background for Eli Lilly by Taconic (Germantown, Maryland, USA), licensed from the Mayo Clinic (Jacksonville, Florida, USA), and imported into the United Kingdom for study at the UCL Centre for Advanced Biomedical Imaging. Mice were kept in individually ventilated cages in groups of 3–5, with ad libitum access to food and water. Animal weight was monitored upon arrival and until experimentation, and all animals were deemed healthy prior to experiments.

For this study, import of 10 female 7.5 month of age rTg4510 and 10 female litter matched wildtype mice occurred two weeks prior to initial imaging studies and subsequent sacrifice, perfuse fixation, and sectioning for immunofluorescent analysis. All the experiments were performed in accordance with the Animals (Scientific Procedures) Act 1986 (ASPA) revised according to the European Directive 2010/63/EU and the UK Home Office (Scientific Procedures) Act (1986) with prior project approval from UCL’s internal Animal Welfare and Ethical Review Body.

### Magnetic resonance imaging of mice

#### Image acquisition

All imaging was performed with a 9.4 T VNMRS horizontal bore scanner (Agilent Inc). A 72 mm inner diameter volume coil (Rapid Biomedical) was used for radiofrequency transmission, and signal was received using a 4-channel array head coil (Rapid Biomedical). Mice were placed in an induction box before anaesthesia was induced using 2% isoflurane at 1 L/min in 100% O_2_. Mice were subsequently positioned in an MRI-compatible head holder to minimize motion artefacts. Anaesthesia was maintained throughout imaging using 1.5% (± 0.2%) isoflurane at 1 L/min in 100% O_2_ delivered via a nose cone, which permitted spontaneous breathing of the mice. Core temperature and respiratory rate were monitored using a rectal probe and pressure pad, respectively (SA Instruments). Mice were maintained at ∼37 °C using heated water tubing and a warm air blower with a feedback system (SA Instruments). Respiration rate was maintained between 80 and 120 breaths per minute by manually adjusting the isoflurane vaporizer.

A 3-dimensional T2-weighted fast spin echo sequence was employed for structural imaging with the following parameters: field of view (FOV) = 19.2 × 16.8 × 12.0 mm, resolution = 150 × 150 × 150 μm, repetition time (TR) = 2500 ms, effective echo time = 43 ms, echo train length = 4, number of signal averages = 1, and imaging time = 1.5 h.

Mice were imaged in a random order, working through cages which housed a mixture of rTg4510 and litter matched wildtype mice.

#### Image analysis

For manual segmentation of the optic nerve and eye structures for extraction of volumes and signal intensities on T2-weighted images, ITK-SNAP segmentation software (v3.x) [49] was used. For extraction of brain volumes, a multi-atlas-based structural parcellation framework was used [25]. Using this framework, we extracted 4 structures of interest to the visual system and dementia: the cortex (containing the visual cortex), thalamus (containing the lateral geniculate nucleus), hippocampus, and superior colliculus. The lateral geniculate nucleus and superior colliculus were of interest as in both humans and mice they are key targets of optic tract axons, and contribute to visual processing, as well as acting as a relay to other destinations in the visual pathway. In addition, we extracted the volume of the whole brain for normalisation purposes. For this, brain images were oriented, non-uniformity corrected, and skull stripped. We adopted the publicly available in vivo mouse brain MRI atlas previously published for the framework [26]. First, the atlas images were registered affinely to the original MR image data using a block-matching algorithm [36]. Once complete, the STAPLE algorithm [25] was applied to fuse the resampled atlas masks together to create a consensus brain mask for each animal’s scans. A further non-rigid registration based on fast free-form deformation was then performed to correct any remaining local misalignment of the affinely registered atlas to the brain volumes [30]. The structural labels from the atlas were then transformed and resampled to the image space of the brain scans using the same affine and non-rigid transformation and fused using the STEPS label fusion algorithm [22] to create the final parcellated structures of interest: the cortex, thalamus, hippocampus, and superior colliculus. A previously published calibration protocol was used to correct gradient scaling errors in the data [32]. Both absolute volumes and volumes normalised to the extracted whole brain volume are presented.

### CSF extraction, perfuse fixation and tissue preparation for histology

Following in vivo MR imaging, animals were terminally anaesthetised with an overdose of Euthanal administered via intraperitoneal injection. A midline incision was made at a midpoint between the skull base and the occipital margin to the first vertebrae. The underlying muscles were parted to expose the atlanto-occipital membrane and dura mater overlaying the cisterna magna. The area was cleaned and a durotomy performed with a 23-gauge needle, allowing CSF to be collected using a narrow bore pipette tip. The thoracic cavities were then opened and the animals were intracardially perfused through the left ventricle: first with 15–20 mL of saline (0.9%) and heparin; second with 50 mL of Formalin, at a flow rate of 3 mL per minute. Following perfusion, the animal was decapitated, de-fleshed, and the head stored at 4 °C and soaked in Formalin for 9 weeks. Brains were removed from the skull and processed using the Tissue TEK VIP processor (GMI Inc.) and embedded in paraffin wax. 6 μm thick sections of the brain in the sagittal plane were collected using a rotary microtome and mounted on glass slides for immunohistochemistry. The eye globes were enucleated, processed (Leica ASP3005) and embedded in paraffin in an orientation that facilitated sectioning in transverse planes. Ice cooled paraffin tissue blocks were sectioned (Leica RM2235 Microtome using S35-PFM feather microtome blades) to generate 4 μm sections which were then placed in a 45 °C water bath. Once mounted onto Super-Frost Plus slides, sections were drip-dried for 2–5 min before heat fixing for 60 mins. Haematoxylin and Eosin (H&E) staining (Autostainer Leica CV5030) and light microscopy (Olympus multi-head light microscope with Micropixx camera software) enabled semi-quantitative morphometric analysis (Image J) and identification of each sections anatomical location in the retinal peripheral-central axis. Sections within 25 μm of the optic nerve head were subsequently used for immunohistochemical staining. The central and peripheral retina were defined as the most lateral and central non-fragmented region of each tissue section (Additional file 1: Figure S1). H&E derived semi-quantitative data included Retinal Ganglion Cell Layer (RGCL) nuclear densities and Inner Nuclear Layer (INL) nuclear densities (number of nuclei occupying a measured area of RGCL or INL respectively), and Inner Plexiform Layer (IPL) thickness relative to total retinal thickness. This data was generated for peripheral and central retinal regions.

### Quantification of CSF tau

The collected CSF was centrifuged briefly to collect any red blood cell contaminates, the supernatant removed and frozen at − 20 °C until further analysis by ELISA. 2 μl water was added to the blood pellet and snap frozen on dry ice to ensure hypotonic freeze-thaw release of haemoglobin from any red blood cells present. Each sample was measured at 417 nm on a NanoDrop spectrophotometer to quantify percentage blood contamination of each CSF sample. This method allows for measurement of blood contamination down to 0.0001%, well below that detectable by eye (~ 0.01%). Lack of significant blood contamination was verified by an average percentage contamination of 0.003% (±0.0006%). Tau content of CSF samples were quantified using both the Human Tau (total), and Human pTau [pS_199_] ELISA Kits (Invitrogen, UK) as per the manufacturer’s instructions.

### Immunohistochemistry

For immunohistochemistry in the retina, retinal sections from 5 rTg4510 and 4 wildtype mice were deparaffinised and hydrated by sequential immersion in xylene, 3 successive ethanol solutions (100, 90, and 70%) and distilled water. As tau proteins largely have intracellular distributions, permeabilisation of nuclear and plasma membranes was carried out using detergents: Tween-20 (0.05%: Sigma-Aldrich P1379) in citrate buffer (Citric acid, anhydrous, Sigma-Aldrich C1857-100G), and Triton-X-100 (0.1%: Sigma-Aldrich T8787-50ML) in phosphate buffered saline (PBS: Gibco 20,012–019) forming PBST. Antigen retrieval was achieved through heating sections in a microwave (setting to 800 W) in citrate buffer (pH 6.0) for 5 mins before slides were left to gradually cool to room temperature (RT) for 20 mins. Following two 5 min PBS washes, sections were blocked with goat serum (Sigma-Aldrich, G9023-10 M: 1:20 in PBST) for 1 h at RT. After performing two 3 min PBST washes sections were incubated with primary anti-pTau (Thermo Scientific MN1020, mouse monoclonal AT8: 1:100 dilution in PBST) and anti-Tau (Dako, rabbit polyclonal A0024: 1:2000 dilution in PBST) antibodies for 1 h at RT. A0024 binds to total tau independent of phosphorylation (amino acids 243–441) whereas AT8 binds to aberrantly phosphorylated tau species (pS_202_/pT_205_). Negative controls, where no primary antibody incubation was performed, were conducted alongside each experimental run (Additional file 1: Figure S2). Following three washes with PBST (5 mins each) sections were incubated with fluorophore conjugated complementary secondary antibodies at RT for 1 h (Alexa-Fluor 568 goat anti-mouse A21124 and Alexa-Fluor 647 goat anti-rabbit A21244, Life Technologies: 1:200 dilution in PBST). This, and all subsequent stages were conducted in the dark to prevent photobleaching. Sections underwent three further 5 min PBST washes, and were then incubated with Hoechst stain (16.2 mM stock solution diluted to 1:5000 in PBS) for 15 mins at RT to provide blue nuclear staining. Two 3 min PBS washes followed. Slides were coverslipped (Mansel-Gläser 24 × 60 mm) following the application of Vectashield anti-fade mounting medium (Vector Laboratories, H-1000) and stored at 4 °C until imaged. Fluorescent confocal microscopy (Zeiss LSM510 confocal microscope with Zen 2 software) generated qualitative and semi-quantitative data. Constant standardised settings were used for comparative non-adjusted imaging, and optimised settings used for adjusted imaging. This semi-quantitative data included the number of pTau positive cells in the RGCL and INL, and the mean intensity per pixel of areas of individual retinal strata (RGCL, IPL, INL and photoreceptor inner segment (PIS)). Data was generated by using Zen & Image J softwares to analyse images of central and peripheral retinal regions. This data, like the semi-quantitative data acquired from H&E images, allowed comparison between rTg4510 and wildtype retinas, and between the central and peripheral retina.

For immunohistochemistry in the brain, brain sections were deparaffinised and hydrated by sequential immersion in xylene, 3 successive ethanol solutions (100, 90, and 70%) and distilled water. Antigen retrieval was performed using the Lab Vision PT module system (Thermo Scientific), where sections were heated to 100 °C for 20 min in citrate buffer (TA-250-PM1X; Thermo Scientific). Slides were transferred to a Lab Vision Autostainer (Thermo Scientific) where the following incubations were performed: 10 min in H_2_O_2_ (0.3%); 30 min in normal goat serum (1:20; Vector Laboratories); 60 min in primary antibody for aberrantly phosphorylated (pS_409_) tau (PG-5; 1:8000 from Peter Davies, Albert Einstein College of Medicine, NY, USA); 30 min in biotinylated goat anti-mouse IgG (1:200, BA9200; Vector Laboratories); 30 min avidin-biotin complex solution (PK-7100; Vector Laboratories); 5 min in 3,3′-diaminobenzidine (SK-4105; Vector Laboratories). Apart from the last two steps, PBST was used for diluting reagents and washes between steps. Sections were then counterstained with haematoxylin before dehydration and cover-slipping. To quantify PG-5-positive tau pathology, stained sections were digitised using the Scanscope AT slide scanner (Aperio) at 20× magnification. Image J software was used to view the digitised tissue sections, threshold for immunoreactivity and delineate boundaries of the visual cortex, hippocampus, thalamus, superior colliculus and optic tract. Data is expressed as the percentage coverage of immunoreactivity in each region.

### Magnetic resonance imaging of humans

#### Participants

Five male subjects fulfilling the criteria for the diagnosis of behavioural variant FTD [40] and carriers of a mutation in the *MAPT* gene (three 10 + 16 and two R406W mutations) were consecutively recruited from a tertiary referral cognitive disorders clinic at the National Hospital for Neurology and Neurosurgery, London, UK. Five healthy controls were also consecutively recruited (1 female, age range: mean(standard deviation) 62(11) years). Patients’ age: 64(3) years; disease duration: 9(3) years; age at onset: 55(1) years. A full clinical history including ophthalmic history was taken from all patients as part of the standard assessment. Hence the patients (and controls) included in this study were free of any retinal or eye disease which may have confounded the data presented.

#### Image acquisition

Volumetric T1- and T2-weighted MRI was performed in all subjects. MRI scans were acquired on a 3 T scanner (Tim Trio, Siemens) with the following sequences: (i) high-resolution isotropic 3D T1-weighted MPRAGE (sagittal orientation; TR = 2200 ms, TI = 900 ms, TE = 2.9 ms, flip angle = 10°, acquisition matrix = 256 × 256 and spatial resolution = 1.1 mm); and (ii) high-resolution isotropic 3D T2-weighted fast spin echo/SPACE (sagittal orientation; TR = 3200 ms, apparent TE = 105 ms, variable refocusing pulse flip angle to achieve T2-weighting, acquisition matrix = 256 × 256 and spatial resolution = 1.1 mm).

#### Image analysis

Acquired T1-weighted images were initially transformed into standard space by a rigid registration to the Montreal Neurological Institute (MNI305) template. Acquired T2 images were registered to the MNI305 template as previously described [6]. Segmentations of the optic nerve were performed manually on about 12 consecutive coronal slices on co-registered T1- and T2- MRIs using NiftyMIDAS (Centre for Medical Image Computing, UCL: http://cmic.cs.ucl.ac.uk/home/software/), starting from the most rostral slice where the eyeball was no longer visible. The intrarater intraclass correlation (ICC) for the optic nerve segmentation, computed with a two-way random effects model, was 0.85 (95% confidence intervals: 0.50–0.96).

### Statistical analysis

Statistical comparisons between animal groups were performed via either a two-way analysis of variance (ANOVA) followed by post-hoc Bonferoni post-tests for multiple comparisons, or (un)paired t-tests for single comparisons, using GraphPad Prism (v5 for Windows, San Diego, CA, USA. Statistical comparisons between patient cohorts were performed via an ANOVA followed by post-hoc Bonferoni post-tests for multiple comparisons, using GraphPad Prism (v5 for Windows, San Diego, CA, USA).). All individual data points are shown with mean ± SEM for animals/participants in each group.

## Results

### pTau in the neurosensory retina of rTg4510 mice

**Fig. 1:**
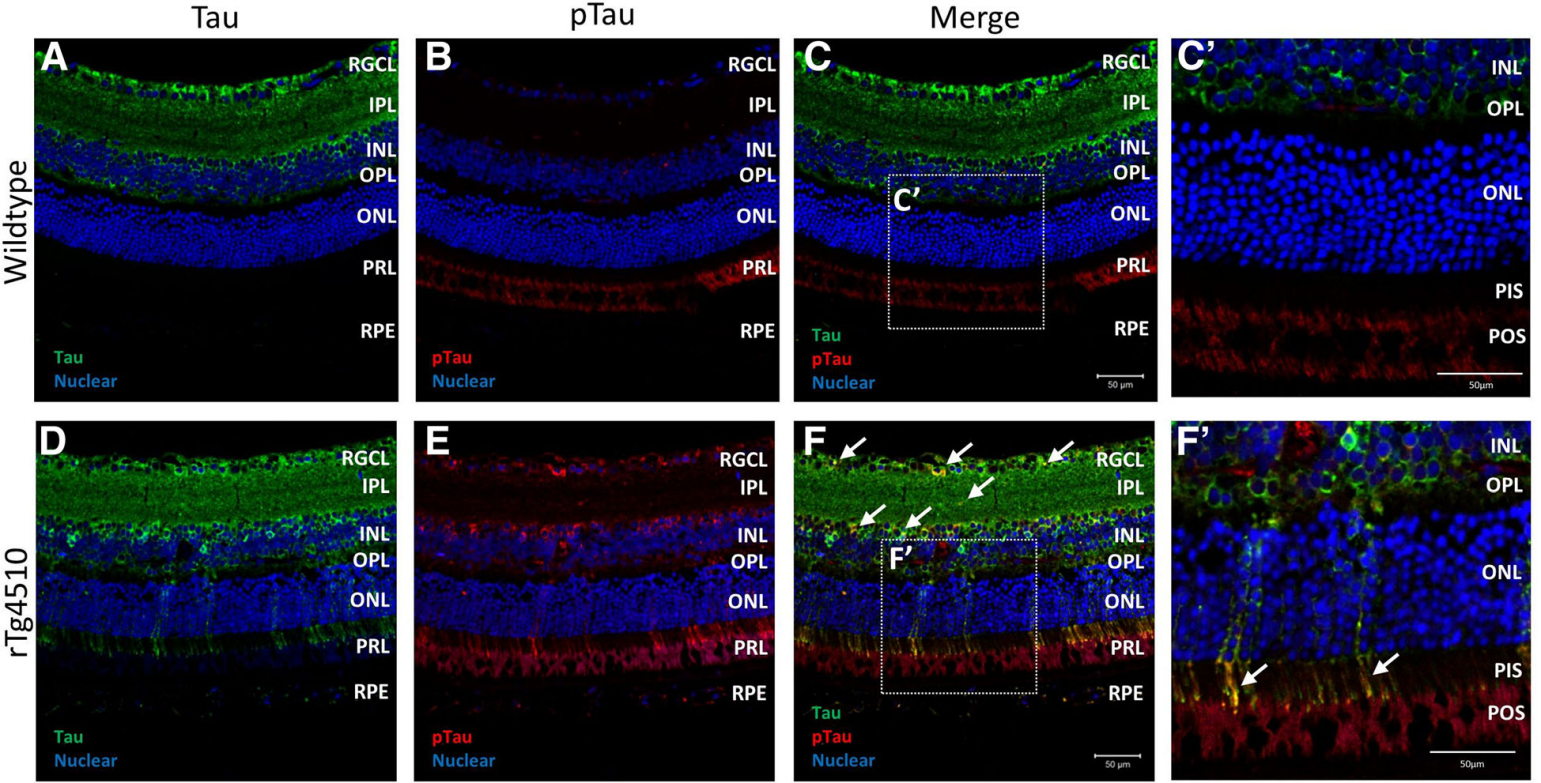
Tau and pTau Immunoreactivity within the Neurosensory Retina of rTg4510 Mice. Representative immunofluorescence images of tau and pTau in the neurosensory retina of wildtype (**a-c**) and rTg4510 (**d-f**) mice. Tau immunoreactivity is shown in green, pTau immunoreactivity is shown in red, and Hoechst nuclear counterstain is shown in blue. Co-localisation of tau and pTau stains indicated with white arrow in merged channel images (**c** and **f**), which can be observed more clearly in higher magnification images (C′ and F′). Scale bars equal to 50 μm

Immunolabelling of total tau with the A0024 antibody in the neurosensory retina of wildtype as well as in rTg4510 mice showed cytosolic and neurite immunoreactivity that was strongest in the retinal ganglion cell layer (RGCL), inner plexiform layer (IPL) and inner nuclear layer (INL) (Fig. 1a and d, respectively). Co-labelling with AT8, which labels pTau, showed occasional weak cell body labelling in wildtype animals (Fig. 1b) that co-localised with total tau (Fig. 1c and C’). The red signal observed in the photoreceptor layer on Fig. 1b was the result of the combination of photoreceptor outer segment (POS) autofluorescence and nonspecific secondary antibody binding that did not differ from what was observed in control sections to which no primary antibody was added (Additional file 1: Figure S2). In contrast, in the rTg4510 animals, numerous neuronal somas and neurites were pTau immunopositive in the RGCL, IPL and INL (Fig. 1e) that showed co-localisation throughout, with more intense total tau labelling (Fig. 1f, white arrows) and irregular clusters extending to the PIS were also observed (Fig. 1F’, white arrows).

**Fig. 2:**
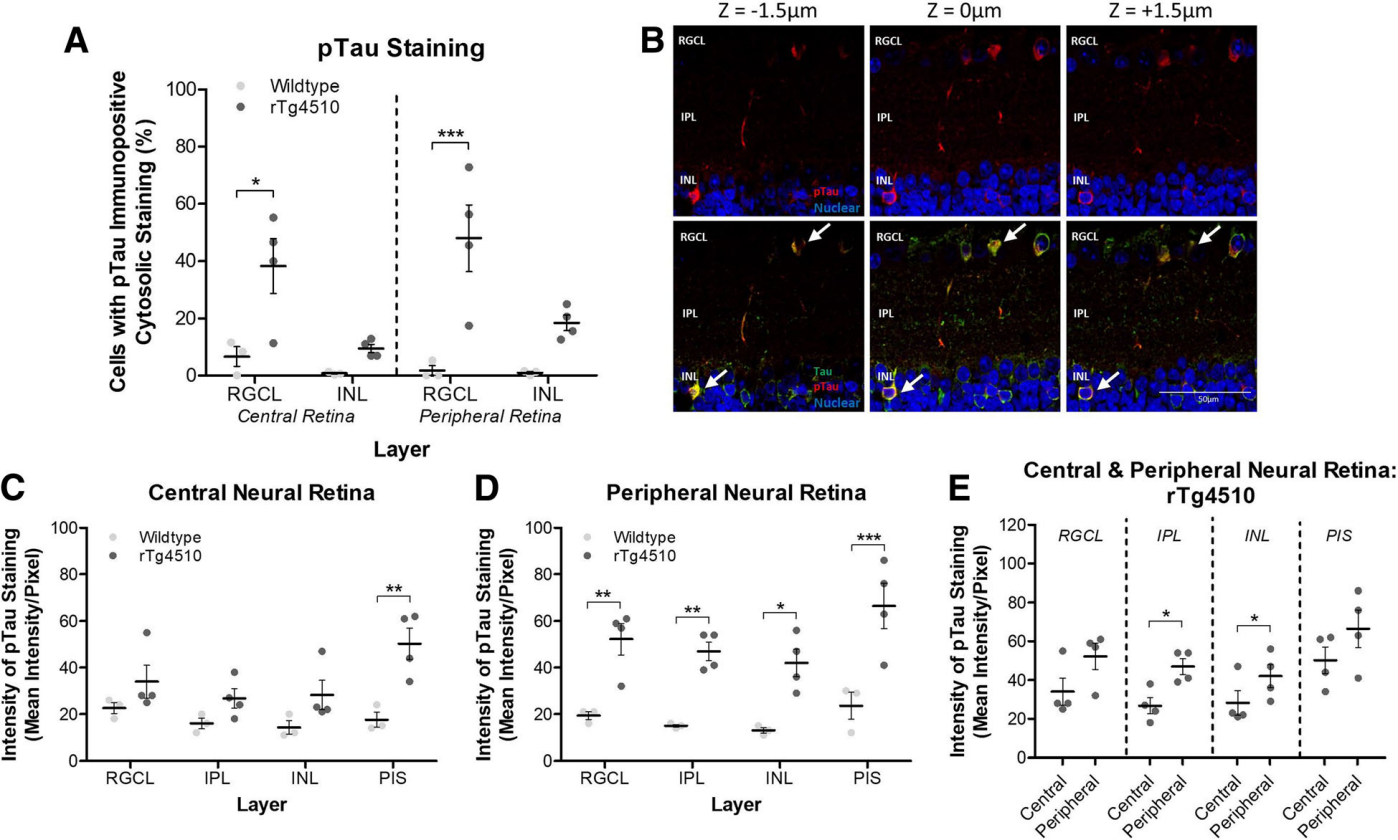
Quantification of Tau and pTau Immunoreactivity in the Neurosensory Retina of rTg4510 Mice. **a** Normalised (to total cells analysed) quantification of the percentage of cells displaying with pTau immunopositive cytosolic staining in the Retinal Ganglion Cell Layer (RGCL) and Inner Nuclear Layer (INL) of the central and peripheral retina. Two-way ANOVA (*n* = 3/4, F_1,3_ = 31.78, *p* < 0.0001). **b** Confocal z-stacked images demonstrating the flame-shaped morphology of cytosolic pTau inclusions in the RGCL and INL (indicated by white arrows) and bead-like neurite inclusions. Scale bars equal to 50 μm. Immunoreactive intensity (intensity per image pixel) of pTau in the RGCL, Inner Plexiform Layer (IPL), INL, and inner segment of the Photoreceptor Layer (PIS) in the (**c**) central neurosensory retina (two-way ANOVA (n = 3/4, F_1,3_ = 20.08, *p* = 0.0002)) and (**d**) peripheral retina (two-way ANOVA (*n* = 4, F_1,3_ = 63.03, p < 0.0001)). **e** Comparison of immunoreactive intensity between the central and peripheral regions of the neurosensory retina in the RGCL, IPL, INL and PIS layers of the rTg4510 mice (paired t-tests). Statistical significance indicated with asterisks: * = *p* < 0.05; ** = *p* < 0.01; *** = *p* < 0.001

Quantification of the number of cell bodies positive for pTau as a percent of total cell nuclei numbers that were counted as Hoechst positive labelling, showed a significantly higher proportion of pTau immunopositive cells in the RGCL of the central (*p* < 0.05) and peripheral (*p* < 0.001) retinas of rTg4510 compared to wildtype mice (Fig. 2a). Upon examination of several serial confocal images (z-stacks), pTau and tau inclusions in the RGCL and INL were shown to have morphologies reminiscent of flame-shaped inclusions of tangle-like tau (Fig. 2b, white arrows). When comparing overall average pixel intensities in pTau immunofluorescence in the central retinas between wildtype and rTg4510 mice, we found significantly higher labelling in the PIS (*p* < 0.01), but no significant difference in RGCL, IPL and INL in the transgenic animals (Fig. 2c). However, when the peripheral retina was compared, the RGCL, IPL INL, and the PIS, showed significantly greater pTau burden in the rTg4510 compared to the wildtype mice (p < 0.01, p < 0.01, *p* < 0.05 and *p* < 0.001 respectively) (Fig. 2d). When comparing pTau immunofluorescence intensities between the central and peripheral retina of the rTg4510 mice, there was a trend towards higher intensities in peripheral retina of all layers but only the IPL and INL showed statistically significant higher pTau staining intensities (both p < 0.05) (Fig. 2e). Lastly, in rTg4510 retinas stained with pTau and total tau, labelling was consistently seen in axons of the RNFL (Fig. 1f), which ultimately amass to form the optic nerve.

### Decrease in RGC densities in rTg4510 mice

**Fig. 3:**
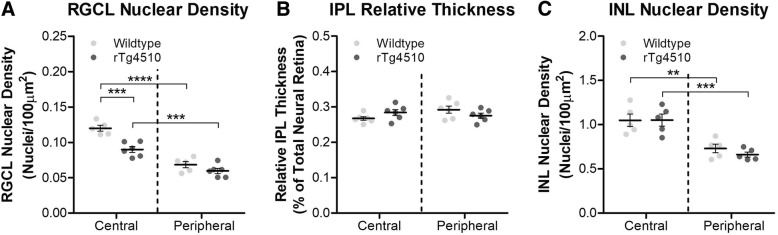
– Neurodegeneration Within Layers of the Neurosensory Retina of rTg4510 Mice. **a** Differences in nuclear density (nuclei per tissue area) in the Retinal Ganglion Cell Layer (RGCL) of the central and peripheral retina of wildtype and rTg4510 mice. Two-way ANOVA (*n* = 6, F_1,1_ = 22.73, p = 0.0002). **b** Relative thickness (% of total neural retina) of the Inner Plexiform Layer (IPL) of the central and peripheral retina of wildtype and rTg4510 mice, as ascertained from H&E staining. Two-way ANOVA (n = 6, F_1,1_ = 0.00002853, *p* = 0.9958). **c** Differences in nuclear density (nuclei per tissue area) in the Inner Nuclear Layer (INL) of the central and peripheral retina of wildtype and rTg4510 mice. Two-way ANOVA (*n* = 6, F_1,1_ = 0.3358, *p* = 0.5704). Statistical significance indicated with asterisks: ** = *p* < 0.01; *** = *p* < 0.001, **** = *p* < 0.0001

The nuclear density of the RGCL was evaluated on H&E stained 4 μm paraffin embedded sections. RGC numbers were significantly reduced in the central but not in the peripheral retina of rTg4510 compared to wildtype animals (*p* < 0.001) (Fig. 3a). The RGC numbers were lower in the periphery compared to the central retina in both wildtype and rTg4510 animals (p < 0.001) (Fig. 3a). When comparing the thickness of the IPL, no differences between the central and peripheral retina, nor between animal groups was observed (Fig. 3b). No differences were observed in the nuclear density of the INL between wildtype and rTg4510 mice, although there were significantly fewer cells present in the retinal periphery in all mice (Fig. 3c).

### Neurodegeneration in the visual system of rTg4510 mice

**Fig. 4:**
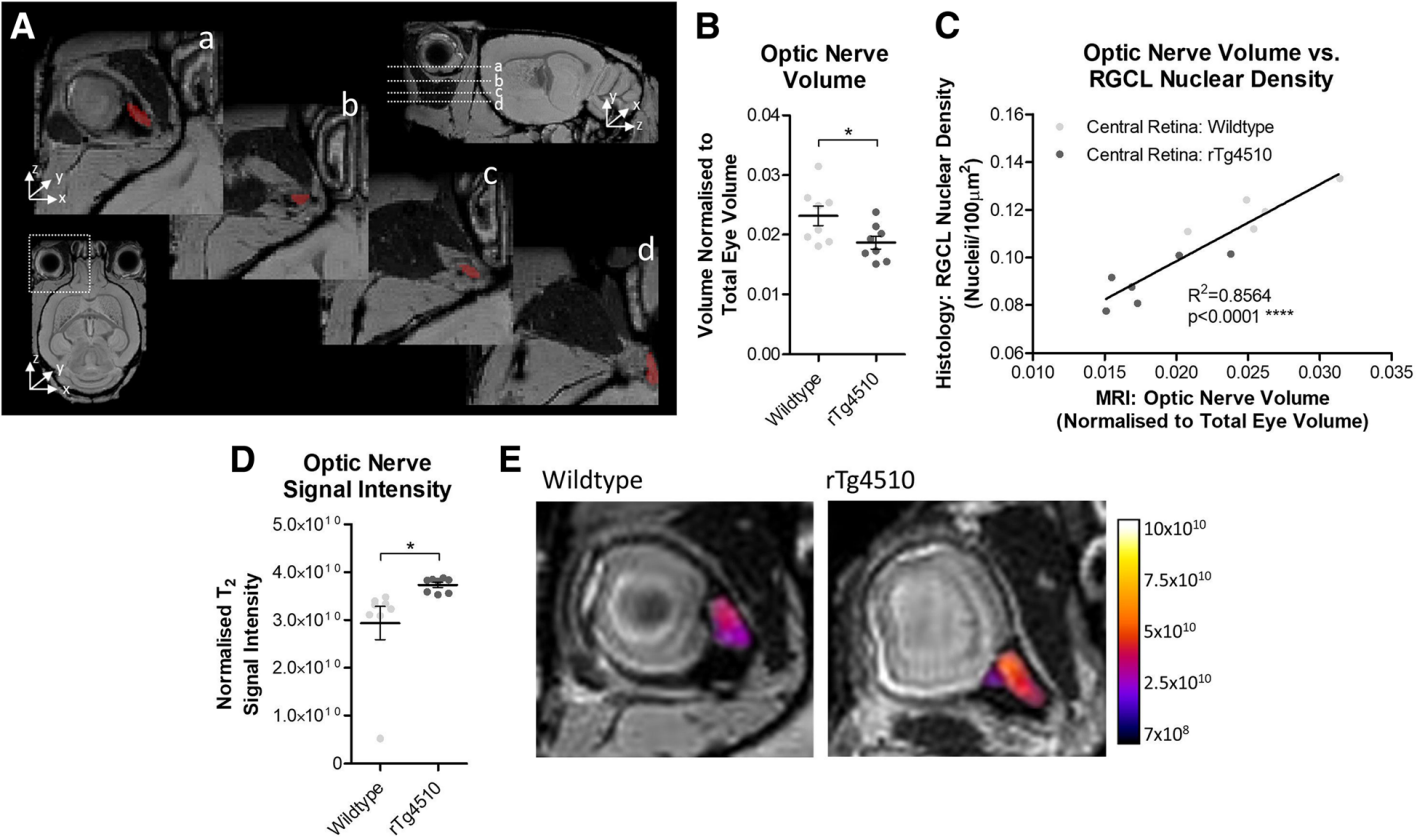
Reduced Optic Nerve Volume in rTg4510 Mice Ascertained Through MRI. **A** Example images demonstrating the segmentation protocol used for volumetric analysis of the optic nerve from T2-weighted MR images. (a, b, c and d) Different horizontal levels of the 3D MR images, with the optic nerve highlighted in red. **B** Normalised (to total eye volume) volume of the optic nerve in wildtype and rTg4510 mice. Unpaired t-test (*n* = 8, *p* = 0.0375). Un-normalised and total eye volumes are shown in Additional file 1: Figure S4. **C** Correlation between MRI derived measures of optic nerve volume, and RGCL nuclear density derived from histology. Linear regression analysis (F_1,9_ = 53.66, p < 0.0001). (**D**) Difference in normalised T2 signal in the optic nerve of wildtype and rTg4510 mice. Unpaired t-test (n = 8, *p* = 0.0393). (**E**) Representative T2-weighted MR images of a cross-section of the optic nerve, with the optic nerve region pseudo colour scaled for signal intensity. Statistical significance indicated with asterisks: * = *p* < 0.05, **** = *p* < 0.0001

To examine whether there were tau induced neurodegenerative changes in parts of the visual system, brain T2-weighted MR images were examined (Fig. 4a). Segmentation of the optic nerve demonstrated a significant reduction in normalised (to total eye volume) optic nerve volume in rTg4510 mice compared to wildtype animals (*p* < 0.05) (Fig. 4b) (individual non-normalised optic nerve and total eye volumes are shown in Additional file 1: Figure S4). We also showed that optic nerve volume was closely correlated with RGCL nuclear density (R^2^ = 0.8564, *p* < 0.0001) (Fig. 4c). In parallel, the optic nerve of rTg4510 mice presented with a greater signal intensity on T2-weighted MR images (*p* < 0.05) (Fig. 4d and e), suggestive of increased water content or oedema within the optic nerve of rTg4510 animals, which is consistent with the hypothesis of axonal degeneration.

**Fig. 5:**
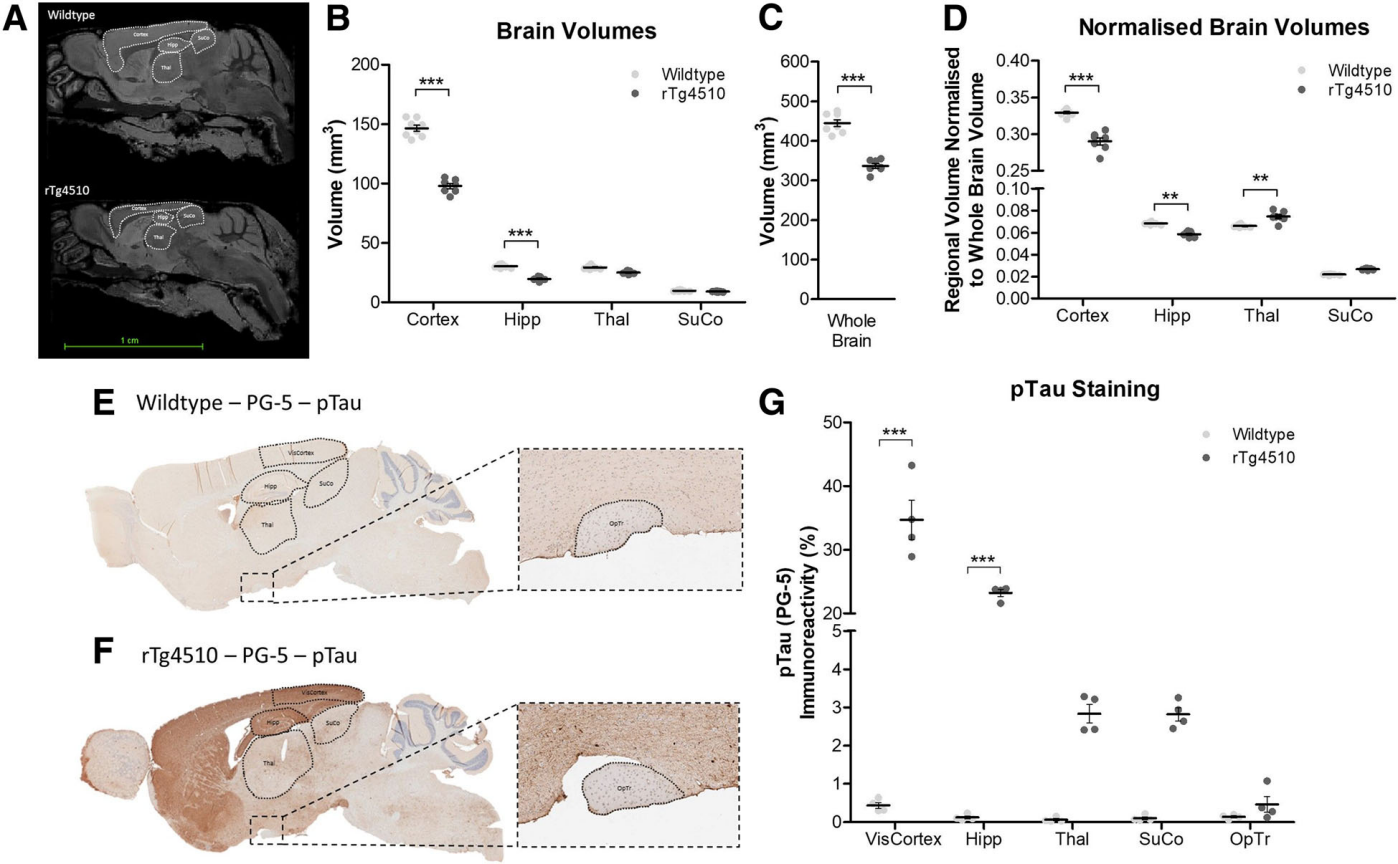
– Tau Deposition and Neurodegeneration of Visual Pathways in the Brains of rTg4510 Mice. **a** Representative MR images of the brains of wildtype and rTg4510 mice displayed in the sagittal plane, with the visual cortex, hippocampus, thalamus and superior colliculus delineated. **b** Volumes of the cortex, hippocampus (Hipp), thalamus (Thal) and superior colliculus (SuCo) as ascertained through automated parcellation of MR images. Two-way ANOVA (*n* = 8, F_1,3_ = 307.7, *p* < 0.0001). **c** Wildtype and rTg4510 animal whole brain volumes extracted from MR images. Unpaired t-test (*n* = 8, *p* < 0.0001). **d** Regional volumes displayed in (**b**) normalised to whole brain volume displayed in (**c**). Two-way ANOVA (n = 8, F_1,3_ = 45.30, p < 0.0001). Representative PG-5 immunoreactivity in the (**e**) wildtype and (**f**) rTg4510 brain, with visual cortex (VisCortex), hippocampus (Hipp), thalamus (Thal), superior colliculus (SuCo) and optic tract (OpTr) delineated demonstrating forebrain localisation of pTau, and for extraction of immunoreactivity data presented in (**g**) pathology in the brain. Two-way ANOVA (n = 8, F_1,4_ = 404.1, *p* < 0.0001). Statistical significance indicated with asterisks: ** = *p* < 0.01; *** = *p* < 0.001

Parcellation of brain regions involved in visual processing and memory from T2-weighted MR images of the mouse brain (Fig. 5a) revealed significant atrophy within the cortex (encompassing the visual cortex) (*p* < 0.001) and the hippocampus (*p* < 0.001) of rTg4510 mice compared to wildtype, but not in the thalamus (encompassing the lateral geniculate nucleus) or superior colliculus (Fig. 5b). Furthermore, when the volumes of these regions were normalised to total brain volume, significant atrophy was still apparent in the cortex (*p* < 0.001) and hippocampus (*p* < 0.01) indicative of atrophy over-and-above that experienced by the whole brain. The thalamus (encompassing the lateral geniculate nucleus) of the rTg4510 mice however exhibited a significantly larger normalised volume than the wildtype mice (p < 0.01), indicative of sparing of this brain region in the rTg4510 mouse in relation to the extent of whole brain atrophy (Fig. 5d). A similar, albeit not significant difference was also observed in the normalised volume of the rTg4510 superior colliculus (Fig. 5d). These observed reductions in volume within the cortex and hippocampus, and sparing of the thalamus in this model were consistent with the localised expression of mutant MAPT gene and deposition of tau pathology in the brain (Fig. 5e and f), indicative of tau induced neurodegeneration. More specifically, significantly greater levels of pTau immunoreactivity were observed in the visual cortex (p < 0.001), and hippocampus (p < 0.001) of rTg4510 mice compared to wildtype animals (Fig. 5g). Subtle increases in the level of pTau staining in the rTg4510 thalamus (encompassing the lateral geniculate nucleus), superior colliculus, and optic tract, were also observed, however these differences were not significant. Tau induced neurodegeneration within the brain of these animals is also consistent with the significant amounts of both total and phosphorylated tau species detected in CSF extracts from rTg4510 mice, but not in wildtype animals (Additional file 1: Figure S5).

### Neurodegeneration in the optic nerve of frontotemporal dementia patients

**Fig. 6:**
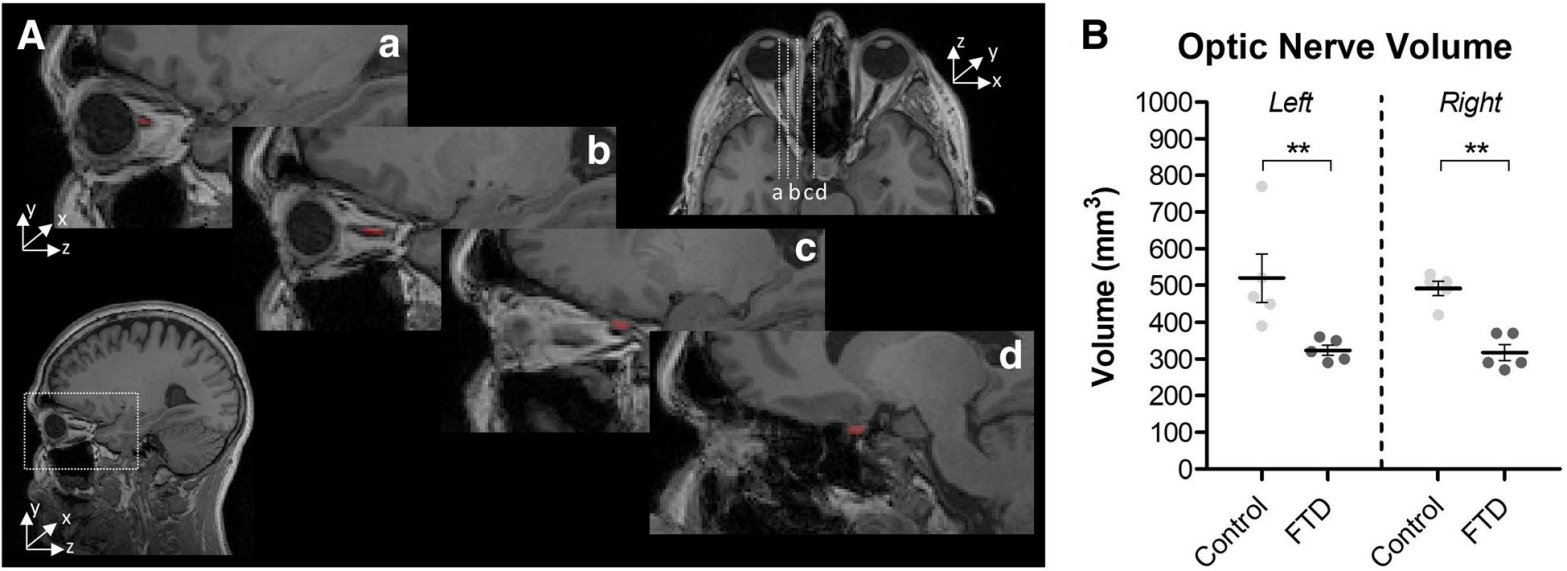
– Reduced Optic Nerve Volume in MAPT Mutation Carrying FTD Patients. **A** Example images demonstrating the segmentation protocol used for volumetric analysis of the optic nerve from T1-weighted MR images. (a, b, c and d) Different sagittal levels of the 3D MR images, with the optic nerve highlighted in red. **B** Volume of the left and right optic nerves of FTD patients and age-matched healthy controls. Two-way ANOVA (*n* = 5, F_1,1_ = 25.57, *p* = 0.0001). Statistical significance indicated with asterisks: ** = *p* < 0.01

Given the specific atrophy observed in the optic nerve, and its relationship with RGCL nuclear density in the rTg4510 mouse model (Fig. 4c), we sought to determine whether such changes also occur in FTD patients. The volumes of the left and right optic nerves were manually segmented on co-registered T1- and T2-weighted brain MR images of *MAPT* mutation carrying FTD patients, and age matched controls (Fig. 6a). Segmentations revealed a significantly smaller volume of the optic nerve in FTD patients compared to age matched controls (*p* < 0.01, in both left and right) (Fig. 6b). Moreover, these significant differences persisted after optic nerve volumes were normalised to total intracranial volume (Additional file 1: Figure S6), indicative of atrophy over-and-above that experienced by the whole brain in these patients (*p* < 0.01, in both left and right) (Additional file 1: Figure S6B).

## Discussion

The overall finding of this study was that the visual disturbances in FTD sufferers might be, at least partly, due to tau induced degeneration in the neurosensory retina and optic nerve. We demonstrated this through detailed study of the eye and optic nerve of the rTg4510 mouse model of FTD and compared observed changes to those in the brain of the same animal. We also found that changes observed in the mouse optic nerve were similarly observed in human FTD patients, suggesting that the optic nerve and the neurosensory retina may be prone to tauopathic changes in FTD, and inclusion of retinal and optic nerve examination in FTD should be considered in the future.

In the rTg4510 mouse eye, hyperphosphorylated tau pathology was observed mainly in the RGCL, IPL, and INL, with additional irregular clusters within the inner segment of the photo receptor layer (PRL), translating to a significant reduction of nuclear density in the RGCL. This phenomenon was observed to be more pronounced in the peripheral as opposed to the central retina in most layers. The significance of such anatomical organisation of tauopathy in the retina is yet to be fully understood, however this regional nature of retinal pathology observed here in the rTg4510 eye mirrors the pattern of pathology we observed recently in the retina in AD [11]. The axons of RGCs in this layer converge in the RNFL to form the optic nerve, where we observed an elevated T2 signal, indicative of oedema/demyelination, and a decrease in volume. Unsurprisingly, this optic nerve atrophy was associated with RGCL nuclear density loss. Most interesting however, was that similar optic nerve atrophy was observed in cases of human FTD, suggesting that the optic nerve, and by extension, the RGCL, are prone to tauopathic changes in FTD. The consequence and pathophysiological relevance of such changes depend upon the context of cell populations affected by tauopathy. While the RGCL is composed largely of RGCs, displaced amacrine cells (ACs) are suggested to encompass up to 59% of the total cells in this layer [38]. ACs are distinguishable from RGCs by their smaller soma and lack of projecting axons in the RNFL, hence these cells are unlikely to contribute directly to the optic nerve atrophy observed here. Hence rather, cells affected by the increase in pTau immunoreactivity and decrease in nuclear density in the RGCL are likely to be RGCs in origin. That being said, double staining for pTau and markers of RGCs themselves in this retinal layer in the rTg4510 is required in order to confirm such a hypothesis. The INL-IPL border on the other hand is itself dominated by ACs with around 1.7% of the cell nuclei here belonging to displaced RGCs [37]. Whether the pTau immunopositive cells identified at the INL-IPL border are actually ACs or displaced RGCs needs further investigation. Yet it would appear, at least from data presented here from the rTg4510 mouse model of tauopathy, that RGC and AC cell populations in the neurosensory retina are the most intrinsically susceptible to tau mediated pathology.

The morphology of RGC and AC pTau inclusions observed in our study compare favourably to NFT pathology found in the brains of other mouse models of FTD and human FTD itself. For example, Deters at al. [12], describe the same AT8 positive flame-shaped and bead like structures of pTau observed in our study (Fig. 2b) as NFTs and somatodendendritic tau inclusions, in the mThy1.2.hTau.P301L mouse brain. Similar morphological expression of pTau has also been described in brains of FTD patients carrying the same P301L mutation in the *MAPT* gene [45]. The prevailing hypothesis from the literature is that such cytoplasmic aggregations of pTau are pathophysiological in nature [4], and hence we hypothesise that the pTau pathology observed in the RGCL in the rTg4510 mouse would be detrimental to the neuronal physiology of resident RGCs. Indeed this idea would be consistent with previous findings from Mazzaro et al., [27], in which reduced activity, assessed through the use of a pattern electroretinogram (pERG) was observed in tau laden RGCs in 5 month old mThy1.2.hTau.P301S mice. Further to this we have observed a significant reduction in nuclear density in this cell layer of the rTg4510 retina, and hence this would suggest that the pTau labelling observed in the RGCL in the rTg4510 mouse is capable of inducing neurodegeneration within this region.

From the retina, axons of RGCs project, via the RNFL and optic nerve to the brain. Given the reduced volume of the optic nerve in rTg4510 mice, and the association of this measure with RGCL nuclear density, this data would suggest tau induced neurodegeneration of RGCs in the retina: with both somal and axonal reductions. In the current study we have not addressed as to whether or not the optic nerve itself is burdened by tau pathology, yet we show that the optic nerve, along with being smaller in rTg4510 mice, is associated with a greater T2 signal. T2 signal is influenced by proton transfers, molecular exchange and diffusion of water, and hence an elevated T2 signal is suggestive of increased water content or oedema within the optic nerve of rTg4510 animals, which is consistent with the hypothesis of neurodegeneration within this region. Moreover, due to the nature of the axonal projections from the RGCs within the eye to the brain, the optic nerve contains a dense packed myelin sheath around the axonal fibres [35]. The hydrophobic properties of the lipidic bilayer in myelin restricts molecular motion of protons [5, 29] and hence hypointensity on T2-weighted MR images reflects larger myelin content. The hyperintensity observed on T2-weighted MR images here then would therefore also be consistent with demyelination of the optic nerve in the rTg4510, a phenomenon which has been previously described in white matter regions of the rTg4510 mouse brain [33, 42]. These quantitative MR findings add weight to the suggestion that indeed neurodegeneration of the RGC axons in the optic nerve is taking place in the rTg4510 mouse.

*MAPT* P301L expression in the rTg4510 mouse is CaMKIIa promoter driven and hence it may well be possible that pTau localisation is due to CaMKIIa expression in RGCs and AC populations. That being said, the CaMKIIa expression profile in the mouse neurosensory retina has not been fully elucidated in the literature. In the rat retina, it has been shown that conventionally placed and displaced ACs are CaMKIIa positive [34], and in the primate retina, CaMKIIa expressing RGCs have been shown to exist [7]. It may well be that the specific promoter system employed in this transgenic mouse causes expression of pTau within these cell layers. Indeed, further assessment of tau pathology in the rTg4510 eye should aim to determine whether or not this is the case, through regional and cell specific profiling of CaMKIIa expression in the mouse retina, to understand the contribution of promoter-driven transgene expression in the deposition of pTau pathology in affected population in the mouse neural retina. Curious however, that similar patterns of expression were also noted in both the mThy1.2 and *PRNP* promoter driven P301 mutant mouse lines [14, 18], suggesting the specific vulnerability of these retinal cell layers, rather than promoter driven expression. Likewise, photoreceptors are unlikely to express CaMKIIa, and yet tau and pTau were both observed in this layer in the rTg4510 mouse retina. This raises the intriguing possibility therefore that mechanisms of tau deposition other than promoter-driven expression, may similarly be occurring in the rTg4510 mouse retina.

**Fig. 7:**
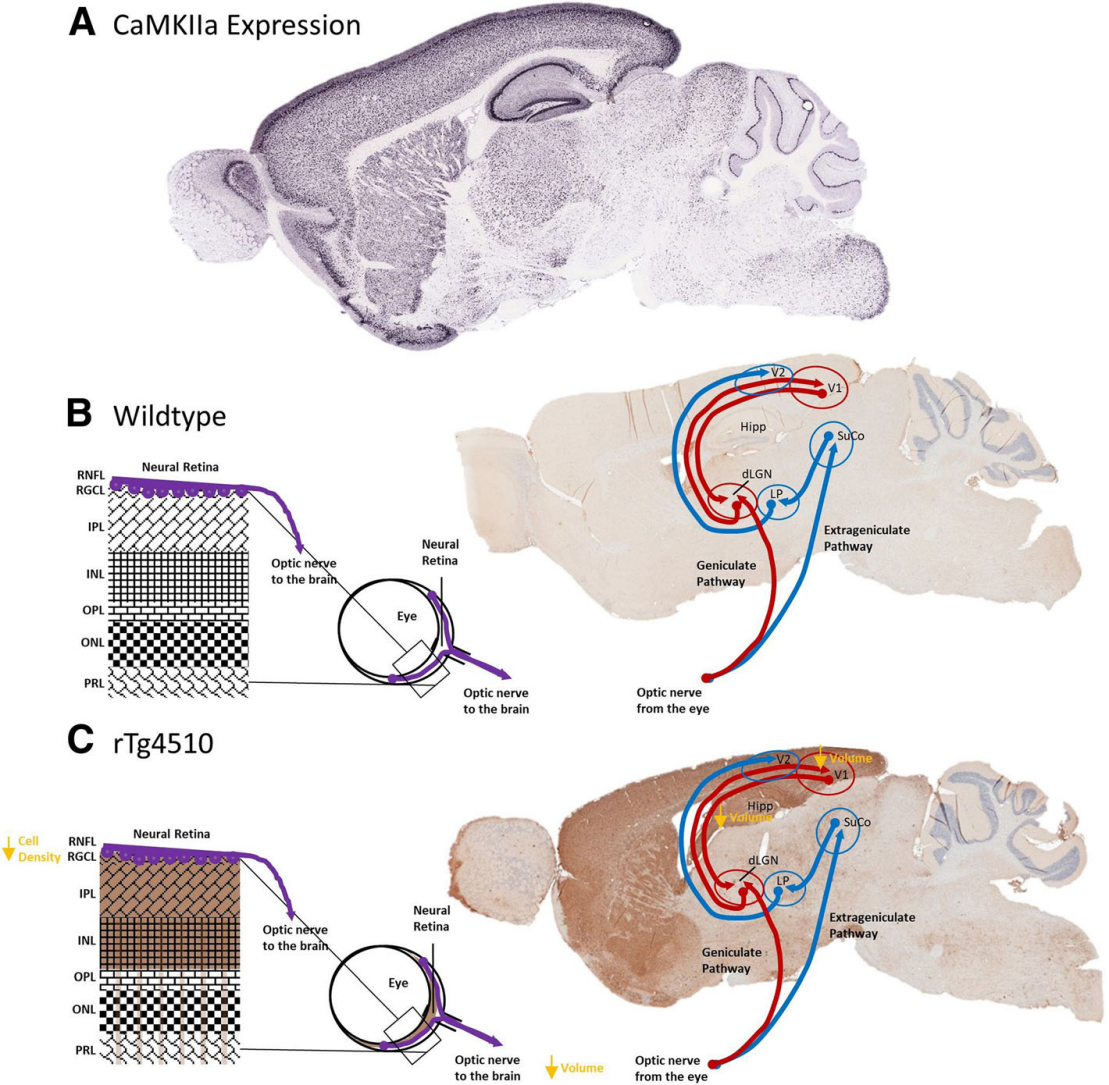
– Summary of Findings of pTau Pathology in the rTg4510 Mouse Visual System. (**a**) Sagittal slice images from the Allen Brain Atlas (Image credit: Allen Institute) showing expression pattern of CaMKIIa in the adult mouse brain. Schematic of the layers of the neural retina, and immunohistochemical slice representative images of tau pathology (brown) in (**b**) wildtype and (**c**) rTg4510 mice, demonstrating the localisation of retinal and parenchymal tau pathology in the brain, overlaid with the geniculate and extra-geniculate visual pathways for contextualisation of the effect of tau pathology on the visual system

It is known that tau propagates in the brain between functionally connected regions [1]. More specific to the visual pathways, it was noted recently by Mazzaro et al. [27], that after tau containing diseased brain homogenate was injected into the Superior Colliculus of mThy1.2.hTau.P301S mice, augmented cerebral, as well as optic nerve pathological tau was evident. The retinal expression pattern of CaMKIIa and promoter-driven tau expression notwithstanding, these data suggest that the retinal tau pathology observed here in the rTg4510 mouse could indeed be a consequence of tau propagation, via the optic nerve, to the retina itself, from functionally connected regions of the visual pathways. As previously mentioned, the expression of the human *MAPT* gene harbouring the P301L mutation in this animal model is driven by the CaMKIIa promoter system (Fig. 7a) and hence tau pathology is observed largely within areas where CaMKIIa is expressed most highly, i.e. the forebrain of mice (Fig. 5f and g), resulting in proportional atrophic changes in brain volume (Fig. 5a-d), as previously observed in this model [47]. Appropriately, we show here that the cortex, including the visual cortex, of the 7.5 month old rTg4510 mouse is heavily burdened by tau pathology (Fig. 5). Yet the subcortical areas involved in visual processing, i.e. the super colliculus and lateral geniculate nucleus, are not overtly affected by either tau pathology and hence nor neurodegeneration, but tau levels appear to be increased compared to controls (Fig. 5g). It is possible therefore, that the tau pathology observed in the retina is a result of propagation from the visual cortex via the geniculate and extra-geniculate visual pathways in the brain (Fig. 7c). This would be consistent with findings of the propagation of tau in the brain from groups such as Ahmed at al. [1], and Mazzaro et al. [27]. Moreover, the incidental finding of the presence of pTau in the PRL, where promoter expression is lacking, would too support, however not prove, the idea that pTau driven pathology in the neurosensory retina is a result of propagation.

In the current study we observed reduced optic nerve volume in FTD patients. Similarly, in a mouse model of the disorder, we demonstrated that reduced optic nerve volume is accompanied by likely optic nerve demyelination (T2 hyperintensity), and associated with a reduction in nuclear density of the tau burdened RGCL. Whether similar levels of neurodegeneration and reduction of RGCL nuclear density take place in the eyes with FTD sufferers will need to be examined in clinical studies, however our finding of reduced optic nerve volume and changes within the neural retina in the rTg4510 mouse model, would recommend assessment of retinal layer thicknesses in FTD. The literature regarding retinal layer thicknesses in FTD patients however is limited and mixed. Specifically, using optic coherence tomography (OCT), Ferrari et al. [13] demonstrate, consistent with the pattern of tau pathology we observed in the mouse eye, NFL and RGCL-IPL layer thinning in FTD compared to healthy controls. Whereas Kim et al. [23], report that there is no difference between inner retinal layer thicknesses of FTD patients compared to control, but that the retinal outer layer is thinner in FTD patients. Interestingly however, a study by Albrecht et al. [2], reveals that patients with another degeneratiuve taupathy, progressive supranuclear palsy, exhibit reduced RGCL, IPL, and outer nuclear layer thicknesses, but greater thickness in the outer plexiform layer compared to control. All the aforementioned studies however only report changes in layer thicknesses rather than any quantitication or localisation of retinal tau pathology. If it is the case though, as supported by the mouse data reported here, that in clinical FTD tau accumulates in layers such as the RGCL leading to RGC neurodegeneration, findings from the glaucoma field would suggest that altered vision is a likely consequence [3]. Together with the atrophic changes taking place in the visual cortex, our findings that the retina and the optic nerve are directly affected by tau pathology may explain the wealth of data linking cognitive visual changes to tauopathies [9, 10, 15, 21, 28] and may suggest that more attention should be paid towards retinal and optic nerve changes in FTD in the clinic. Eye imaging is significantly simpler, faster, and better tolerated than brain imaging. Retinal imaging may therefore serve as a surrogate marker for disease progression, benefiting both patients and the health system enormously.

In conclusion, concurrent degeneration within the neurosensory retina and reduced volume within the optic nerve of rTg4510 mice suggests that tau induced degeneration may play a role in affecting signal propagation to the brain in this animal model. The volume loss of the optic nerve in FTD patients suggests that neurosensory retinal tau pathology may too exist in the eyes of FTD patients, however this would need to be evaluated and confirmed in cadaveric eyes from FTD patients, as well as clinical OCT imaging in FTD sufferers. Currently, the consequences of such pathologies is not known, but preventing and protecting tauopathy patients from the cognitive visual changes routinely experienced with disease development may represent a novel approach for improving quality of life of FTD sufferers, and decrease the burden on carers and society by preserving independent living for longer. Furthermore, it is intriguing to speculate whether optic nerve degeneration could be an early pathological sign of FTD, which could be examined in further longitudinal studies, and which would recommend inclusion of retinal and optic nerve examination in FTD.

## Additional file

Additional file 1: **Figure S1.** Definition of the Central and Peripheral Retina. **Figure S2.** Red Channel Autofluorescence of the Outer Segment of the Photoreceptor Layer. **Figure S3.** Non-normalised Quantification of pTau Immunoreactivity in the Neurosensory Retina of rTg4510 Mice. **Figure S4.** Non-normalised Optic Nerve and Total Eye Volumes of rTg4510 and Wildtype Mice. **Figure S5.** Tau Cerebrospinal Fluid Biomarker in rTg4510 Mice. **Figure S6.** Optic Nerve Volume of FTD Patients Normalised to Total Intracranial Volume. (DOCX 570 kb)
