# Correction to: Optic nerve thinning and neurosensory retinal degeneration in the rTg4510 mouse model of frontotemporal dementia

**DOI:** 10.1186/s40478-019-0780-9

**Published:** 2019-08-05

**Authors:** Ian F. Harrison, Rozalind Whitaker, Pietro Maria Bertelli, James M. O’Callaghan, Lajos Csincsik, Jack A. Wells, Martina Bocchetta, Da Ma, Alice Fisher, Zeshan Ahmed, Tracey K. Murray, Michael J. O’Neill, Jonathan D. Rohrer, Mark F. Lythgoe, Imre Lengyel

**Affiliations:** 1 UCL Centre for Advanced Biomedical Imaging, Division of Medicine, University College London, Paul O’Gorman Building, 72 Huntley Street, London, WC1E 6DD UK; 2 UCL Institute of Ophthalmology, University College London, 11-43 Bath Street, London, EC1V 9EL UK; 3 Dementia Research Centre, UCL Institute of Neurology, University College London, National Hospital for Neurology and Neurosurgery, London, WC1N 3BG UK; 4 Centre for Experimental Medicine, The Queen’s University Belfast, Belfast, BT9 7BL UK; 5 Eli Lilly and Company, Erl Wood Manor, Windlesham, Surrey, UK

**Correction to: Acta neuropathol commun (2019) 7: 4**

**https://doi.org/10.1186/s40478-018-0654-6**

In the original publication of this article [1], the funding acknowledgement for grant “**Alzheimer Society Research Program (ASRP) from the Alzheimer Society of Canada**” was missing. In this correction article the updated funding acknowledgement is shown. The missing funding acknowledgement has been indicated in bold.

The Dementia Research Centre is supported by Alzheimer’s Research UK, Brain Research Trust, and The Wolfson Foundation. This work was supported by the NIHR Queen Square Dementia Biomedical Research Unit and the NIHR UCL/H Biomedical Research Centre, the MRC UK GENFI grant and the Alzheimer’s Society.

IFH is funded by a research grant from Eli Lilly and Company, and the Engineering and Physical Sciences Research Council (EPSRC) UK (EP/N034864/1).

JMO’C is funded by UK Medical Research Council (MRC) Doctoral Training Grant Studentship (MR/J500422/1).

LC is funded by an unrestricted grant from OPTOS Plc. Ltd.

DM received a proportion of funding from EPSRC studentship from UCL’s Centre for Doctoral Training in Medical Imaging, **and a proportion of funding from the Alzheimer Society of Canada through the Alzheimer Society Research Program (ASRP)**.

JDR is supported by an MRC Clinician Scientist Fellowship (MR/M008525/1) and has received funding from the NIHR Rare Disease Translational Research Collaboration (BRC149/NS/MH).

MFL receives funding from BBSRC/AstraZeneca Industrial Partnership Studentship (BB/E528979/1), the UK Regenerative Medicine Platform Safety Hub (MR/K026739/1), Eli Lilly and Company, and the EPSRC (EP/N034864/1).

IL receives funding from the Bill Brown Charitable Trust, Moorfields Eye Hospital Special Trustees and Mercer Fund from Fight for Sight.
