# Supplementary material for: Optic nerve thinning and neurosensory retinal degeneration in the rTg4510 mouse model of frontotemporal dementia
Source: Acta Neuropathol Commun. 2019 Jan 7;7:4. doi: 10.1186/s40478-018-0654-6 (PMC6322294; doi:10.1186/s40478-018-0654-6)
Supplement: Supplementary file 1 — Figure S1. Definition of the Central and Peripheral Retina. Figure S2. Red Channel Autofluorescence of the Outer Segment of the Photoreceptor Layer. Figure S3. Non-normalised Quantification of pTau Immunoreactivity in the Neurosensory Retina of rTg4510 Mice. Figure S4. Non-normalised Optic Nerve and Total Eye Volumes of rTg4510 and Wildtype Mice. Figure S5. Tau Cerebrospinal Fluid Biomarker in rTg4510 Mice. Figure S6. Optic Nerve Volume of FTD Patients Normalised to Total Intracranial Volume. (DOCX 570 kb) [file 40478_2018_654_MOESM1_ESM.docx]

**Optic Nerve Thinning and Neurosensory Retinal Degeneration in the rTg4510 Mouse Model of Frontotemporal Dementia**

**Ian F. Harrison^1^*^†^, Rozalind Whitaker^2^*, Pietro Maria Bertelli^2,4^, James M. O’Callaghan^1^, Lajos Csincsik^2,4^, Martina Bocchetta^3^, Da Ma^1,5^, Alice Fisher^6^, Zeshan Ahmed^6^, Tracey K Murray^6^, Michael J O'Neill^6^, Jonathan D. Rohrer^3^, Mark F. Lythgoe^1ǂ^, Imre Lengyel^2,4ǂ^**

*(1) UCL Centre for Advanced Biomedical Imaging, Division of Medicine, University College London, Paul O’Gorman Building, 72 Huntley Street, London, WC1E 6DD, UK.*

*(2)* *UCL Institute of Ophthalmology, University College London, 11-43 Bath Street, London, EC1V 9EL, UK.*

*(3) Dementia Research Centre, UCL Institute of Neurology, University College London, National Hospital for Neurology and Neurosurgery, London, WC1N 3BG, UK.*

*(4)* *Centre for Experimental Medicine, The Queen’s University Belfast, Belfast, BT9 7BL, UK.*

*(5) School of Engineering Science, Simon Fraser University, 8888 University Drive, Burnaby BC, V5A 1S6, Canada.*

*(5) Eli Lilly and Company, Erl Wood Manor, Windlesham, Surrey, GU20 6PH, UK.*

* *Joint first authors*

^ǂ^ *Joint senior authors*

† Corresponding Author: Dr Ian F Harrison, BSc (Hons) MRes PhD

UCL Centre for Advanced Biomedical Imaging

Division of Medicine

University College London

London, UK

Phone: +44 (0)20 7679 6448

Email: [ian.harrison@ucl.ac.uk](mailto:ian.harrison@ucl.ac.uk)

**Supplementary Material**

**Figure S1 – Definition of the Central and Peripheral Retina**

The central and peripheral retina were defined as the most lateral and central non-fragmented region of each tissue section, delineated and labelled here in a H&E stained section of the retina. Scale bar equal to 500µm.


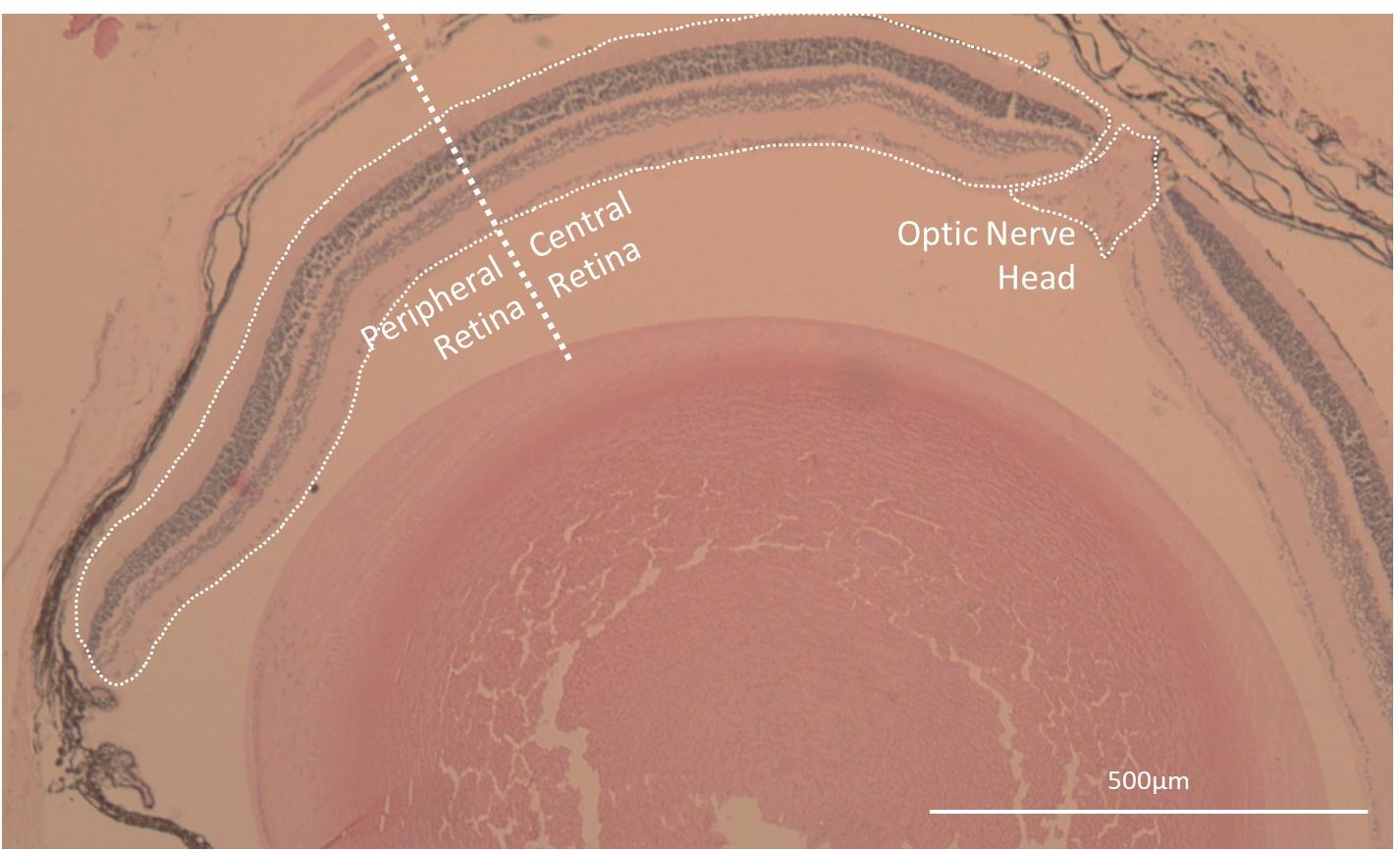


**Figure S2 – Red Channel Autofluorescence of the Outer Segment of the Photoreceptor Layer**

(A) Double labelled immunofluorescence of tau (green) and pTau (red) in a section of the neurosensory retina from an rTg4510 mouse. (B) A tissue section from the same animal stained identically to that displayed in (A) without incubation with either primary antibodies (A0024 or AT8), demonstrating autofluorescence in the red channel of the outer segment of the photoreceptor layer (POS). Scale bars equal to 50µm.


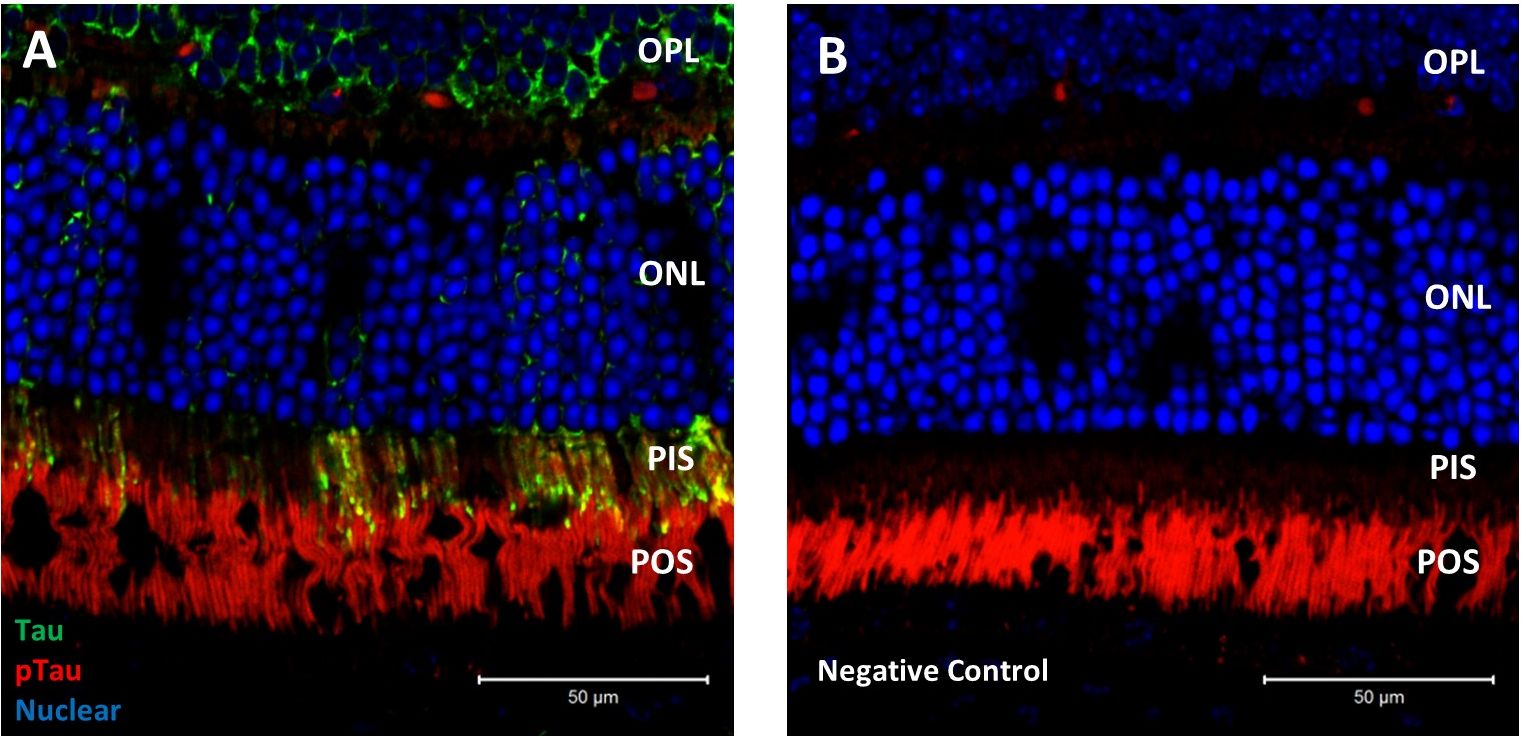


**Figure S3 – Non-normalised Quantification of pTau Immunoreactivity in the Neurosensory Retina of rTg4510 Mice**

Number of cells displaying with pTau immunopositive cytosolic staining in the Retinal Ganglion Cell Layer (RGCL) and Inner Nuclear Layer (INL) of the central and peripheral retina. Two-way ANOVA (F_1,3_=28.26, p<0.0001). Cell counts were normalised to the total number of cells analysed to present as the percentage of pTau cytosolic immunopositivity displayed in figure 2A. Statistical significance indicated with asterisks: **=p<0.01.


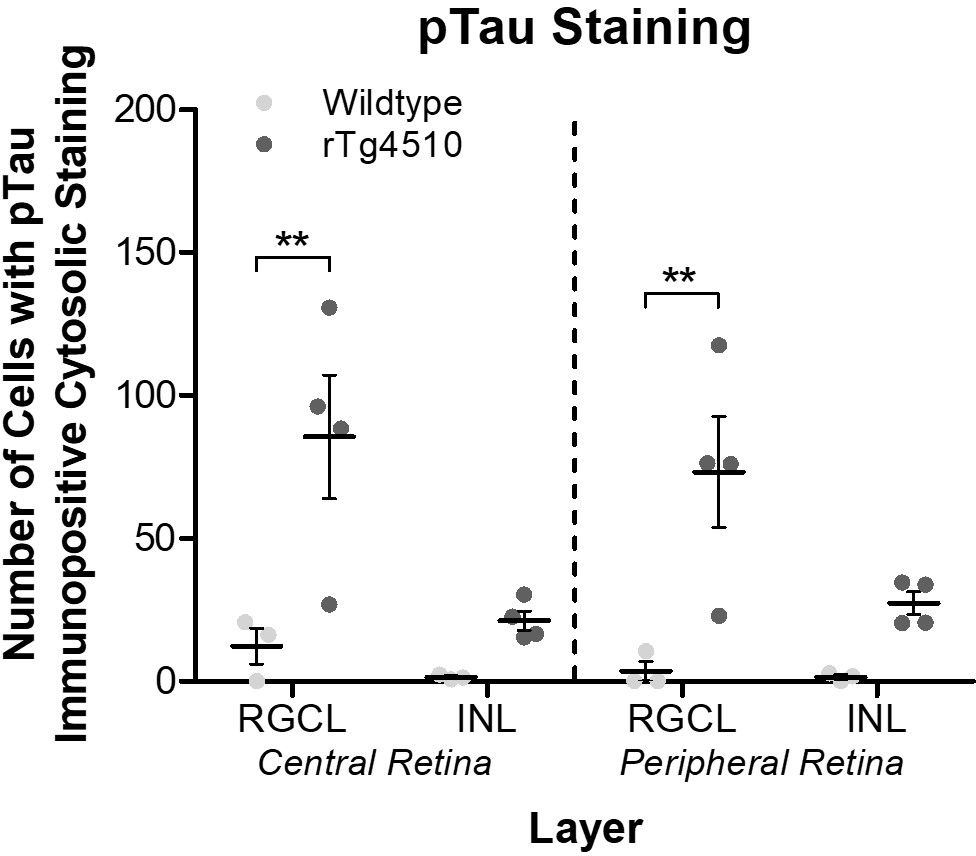


**Figure S4 – Non-normalised Optic Nerve and Total Eye Volumes of rTg4510 and Wildtype Mice**

(A) Individual non-normalised optic nerve volumes extracted from MR images of rTg4510 and wildtype mice, demonstrating the significantly smaller optic nerve of rTg4510 mice compared to wildtype. Unpaired t-test (p=0.0234). (B) Individual total eye volumes extracted from MR images of rTg4510 and wildtype mice showing no difference between animal groups. Unpaired t-test (p=0.3117). Statistical significance indicated with asterisks: *=p<0.05.

**
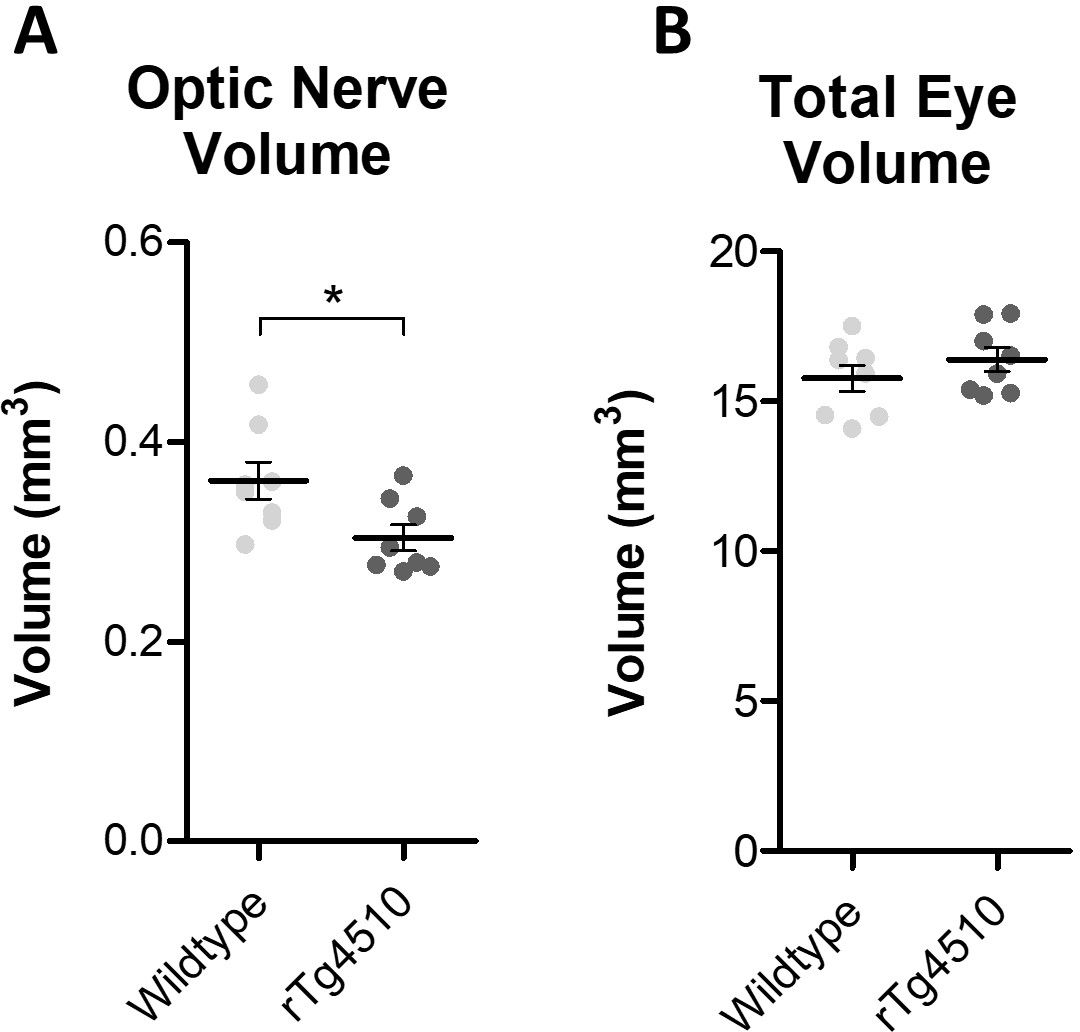
**

**Figure S5 – Tau Cerebrospinal Fluid Biomarker in rTg4510 Mice**

Concentration of tau and pTau in CSF extracted from wildtype and rTg4510 mice demonstrating the elevated presence of both tau species in CSF in the transgenic mouse model. Two-way ANOVA (F_1,1_=55.46, p<0.0001). Statistical significance indicated with asterisks: *=p<0.05; ***=p<0.001.


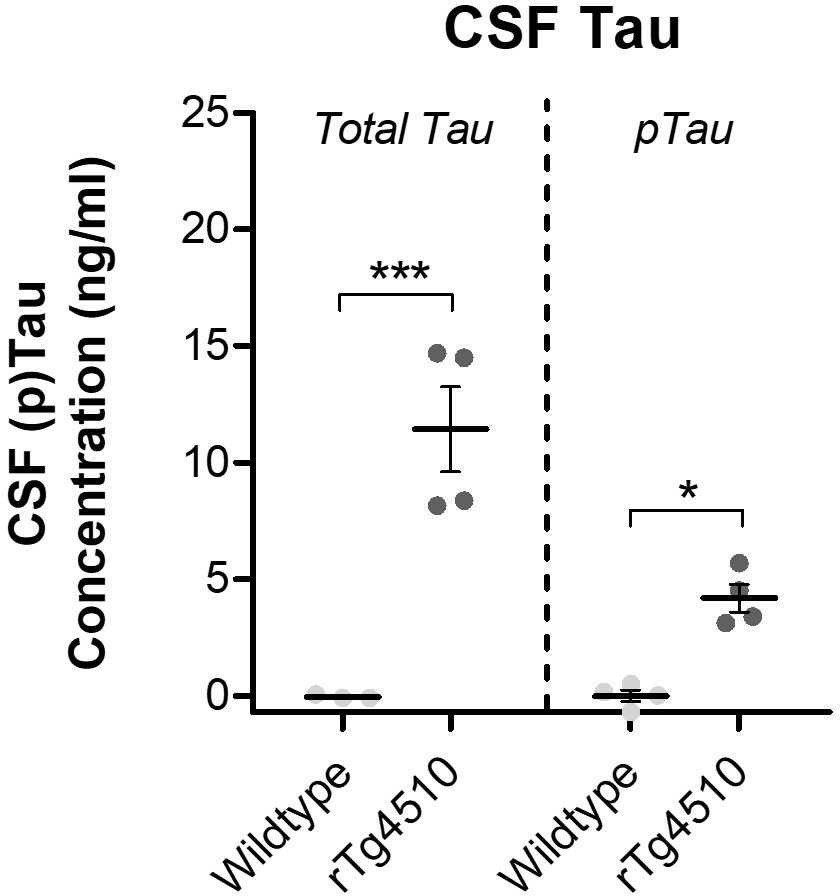


**Figure S6 – Optic Nerve Volume of FTD Patients Normalised to Total Intracranial Volume**

(A) Total intracranial volume of healthy age-matched controls and FTD patients extracted from MR images. Unpaired t-test (p=0.1756). (B) Optic nerve volumes displayed in figure 6B normalised to individual intracranial volumes demonstrating that volume loss is independent of intracranial volume. Two-way ANOVA (F1,1=27.94, p<0.0001). Statistical significance indicated with asterisks: *=p<0.05; **=p<0.01.

**
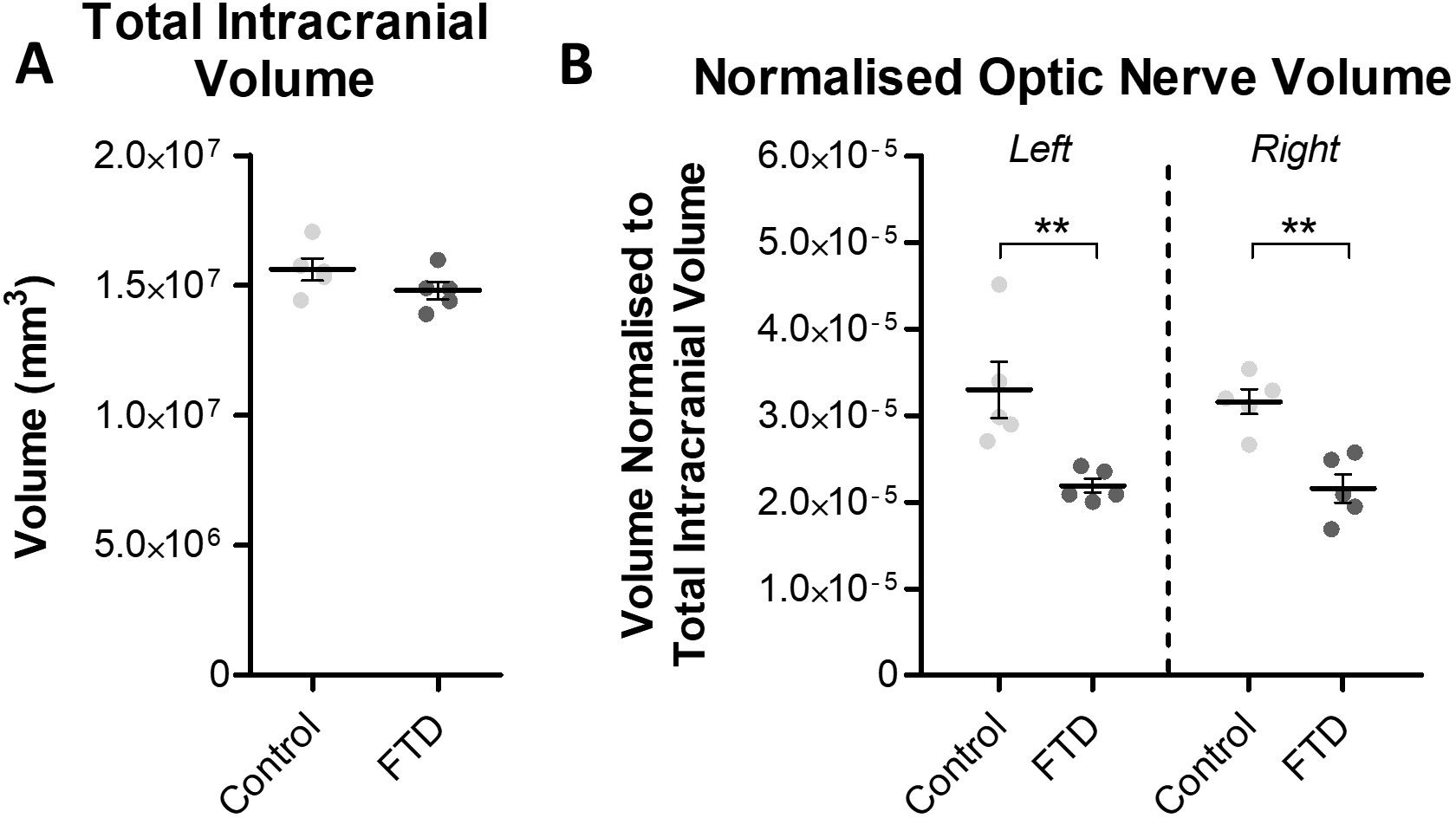
**
